# Distinct microbial communities that differ by race, stage, or breast-tumor subtype in breast tissues of non-Hispanic Black and non-Hispanic White women

**DOI:** 10.1038/s41598-019-48348-1

**Published:** 2019-08-16

**Authors:** Alana Smith, Joseph F. Pierre, Liza Makowski, Elizabeth Tolley, Beverly Lyn-Cook, Lu Lu, Gregory Vidal, Athena Starlard-Davenport

**Affiliations:** 1 Department of Genetics, Genomics and Informatics, Memphis, TN USA; 2 Department of Pediatrics, University of Tennessee Health Science Center, Memphis, TN USA; 3 Department of Medicine, University of Tennessee Health Science Center, Memphis, TN USA; 4 Department of Pharmaceutical Sciences, University of Tennessee Health Science Center, Memphis, TN USA; 5 Department of Preventive Medicine, University of Tennessee Health Science Center, Memphis, TN USA; 6 Division of Biochemical Toxicology, FDA/National Center for Toxicological Research, Jefferson, AR USA; 7 West Cancer Center, Memphis, TN USA

**Keywords:** Bacterial genetics, Breast cancer

## Abstract

Growing evidence highlights an association between an imbalance in the composition and abundance of bacteria in the breast tissue (referred as microbial dysbiosis) and breast cancer in women. However, studies on the breast tissue microbiome have not been conducted in non-Hispanic Black (NHB) women. We investigated normal and breast cancer tissue microbiota from NHB and non-Hispanic White (NHW) women to identify distinct microbial signatures by race, stage, or tumor subtype. Using 16S rRNA gene sequencing, we observed that phylum Proteobacteria was most abundant in normal (n = 8), normal adjacent to tumor (normal pairs, n = 11), and breast tumors from NHB and NHW women (n = 64), with fewer Firmicutes, Bacteroidetes, and Actinobacteria. Breast tissues from NHB women had a higher abundance of genus *Ralstonia* compared to NHW tumors, which could explain a portion of the breast cancer racial disparities. Analysis of tumor subtype revealed enrichment of family *Streptococcaceae* in TNBC. A higher abundance of genus *Bosea* (phylum Proteobacteria) increased with stage. This is the first study to identify racial differences in the breast tissue microbiota between NHB and NHW women. Further studies on the breast cancer microbiome are necessary to help us understand risk, underlying mechanisms, and identify potential microbial targets.

## Introduction

Breast cancer is the most common cancer in women worldwide^1^. In the United States, more than 200,000 new breast cancer cases will be diagnosed this year alone^2^. Of these women, non-Hispanic black (NHB) women are more likely to die from breast cancer compared to other racial/ethnic groups^3^. Additionally, NHB women are more likely to be diagnosed with an aggressive form of breast cancer, known as triple negative breast cancer (TNBC) that does not respond to hormonal breast cancer therapies^4,5^.

The cause for the racial disparities in breast cancer risk and outcomes observed between NHB and NHW women is unclear; however, mounting evidence suggests that an imbalance in the collective genome of microorganisms, referred to as microbial dysbiosis, may be associated with the development of human diseases including cancer^6–8^. More recently, breast tissues have been observed to have their own unique microbiome that is distinct between pathologically defined normal, benign, and malignant breast tissues^6,9–11^. However, these studies did not include NHB women in their analysis.

In the present study, we defined unique microbial signatures in normal breast and breast tumor with paired normal adjacent breast tissue samples obtained from NHB and NHW women using 16S rRNA gene sequencing for the first time. This study highlights that disease state, tumor subtype, race, and stage of breast cancer display an altered microbiome and should be measured in all breast cancer studies to better understand how microbial dysbiosis influences breast cancer development and outcomes in ethnically diverse populations and if biomarkers can be identified to stratify risk or response to treatment.

## Results

### Patient demographic and breast tissue characteristics

We analyzed a total of 83 breast tissue samples, of which pathologically adjacent normal breast tissues (normal pair) were obtained from 11 breast cancer patients (Supplementary Table 1). Table 1 presents the selected demographic and tissue characteristics of patients’ breast tissue samples. A total of 64 breast cancer tissues were collected from women with stage I-IV breast cancer (tumor) and 8 from women who underwent breast reduction mammoplasty (normal). Approximately 24% of the study participants were NHB, 75% NHW, and 64% were premenopausal. The mean age of breast cancer patients in this study was significantly higher than healthy controls (47 ± 1.24 versus 30 ± 3.86, p < 0.0001). A total of 13 stage 1, 24 stage II, and 19 stage III and IV breast cancer tissues were analyzed in this study. We combined stages III and IV breast tumors together due to low number of stage IV breast tumors. Staging information was unavailable for 8 breast cancer tissue specimens. We also analyzed breast cancer tissues by the 4 major breast tumor subtypes: luminal A, luminal B, human epidermal growth factor receptor 2 (HER2), and TNBC^12,13^. Of the breast cancer tissues, 34% were Luminal A, 22% Luminal B, 9% HER2, and 23% TNBC. Tumor receptor status was not available for 7 of the breast cancer tissue specimens.

**Table 1:** Patient breast tissue characteristics.

Variable	Total patients (n = 72)	Normal (n = 8)	^a^Tumor (n = 64)
**Mean Age**, **years**			
Average (range)	45 (18–72)	30 (18–52)	47 (25–72)
**Ethnicity**			
NHB	17	5	12
NHW	54	3	51
Missing	1	0	1
**Menopausal Status**			
Premenopausal	46	7	39
Postmenopausal	24	1	23
Missing	2	0	2
**Stage**			
1	NA	NA	13
2	NA	NA	24
3/4	NA	NA	19
Missing	NA	NA	8
**Tumor Subtype**			
Luminal A	NA	NA	22
Luminal B	NA	NA	14
HER2	NA	NA	6
TNBC	NA	NA	15
Missing	NA	NA	7

^a^The total number of tissue samples used in this study (n = 83) also includes adjacent normal breast tissue samples, labeled normal pairs (n = 11) from the same breast cancer patient.

Abbreviations: NHB: Non-Hispanic Blacks; NHW: Non-Hispanic Whites; TNBC: triple negative breast cancer; NA: not applicable; SD: standard deviation.

### Breast tissue microbiome characteristics in normal and breast tumor tissues

**Figure 1:**
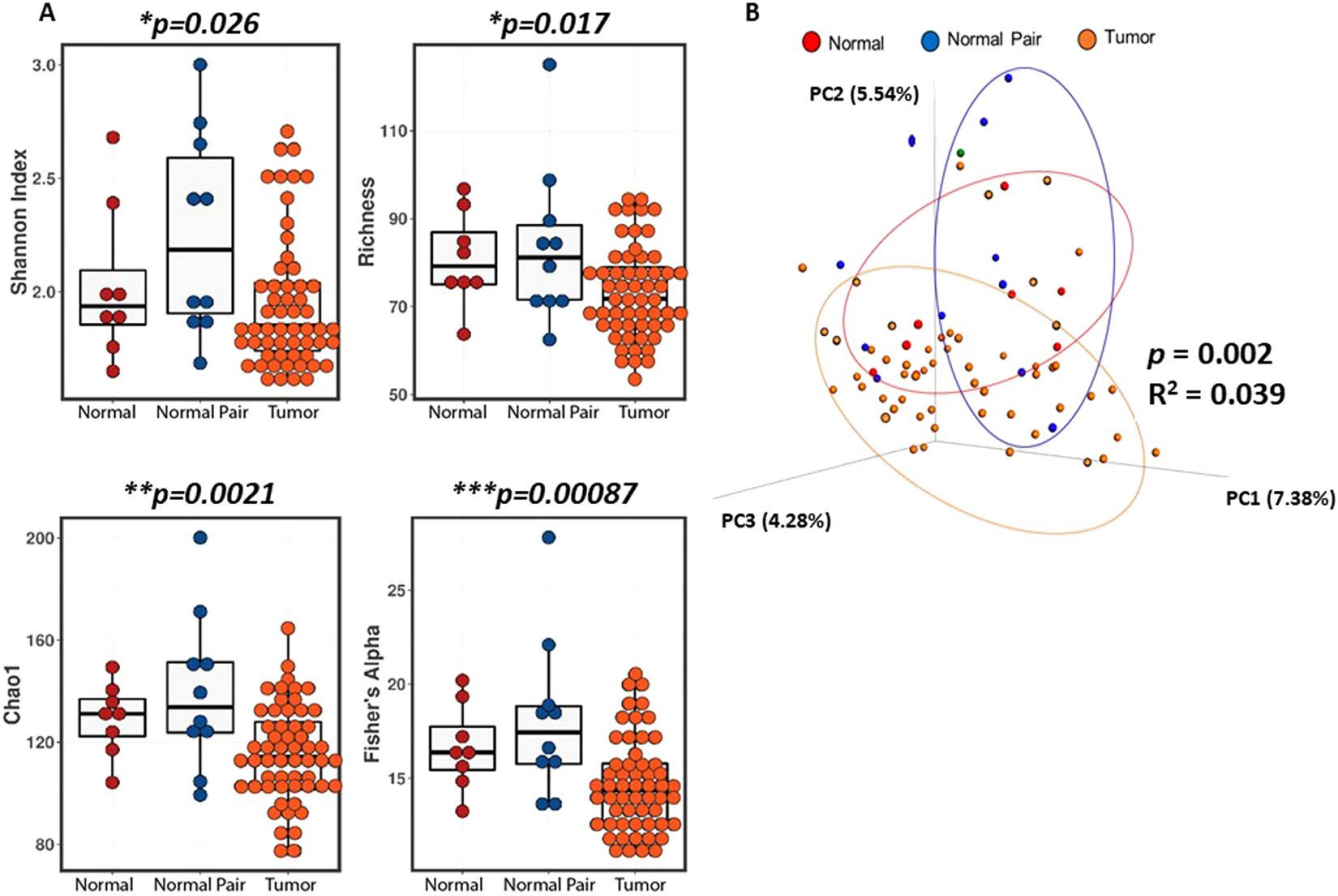
Microbial diversity exists between normal breast tissue, tumor and adjacent normal breast. (**A**) Bar graphs compare the alpha-diversity (Shannon diversity), Richness, Chao1, and Fisher’s alpha measures of read counts between normal (n = 8), normal pair (n = 11), and tumor (n = 64) tissue sample types (Shannon index, *P* = 0.026; Richness, *P* = 0.07; Chao, *P* = 0.0021; Fisher’s alpha, *P* = 0.00087). (**B**) Principal coordinates (PCs) plots show the clustering pattern of the three groups based on unweighted UniFrac distance and is colored by sample types (red circles - normal, blue circles - normal pair, orange circles – tumor samples); *P* = 0.002 and R^2^ = 0.039.

The Shannon index between normal, normal pair, and tumor disease states was significantly different, *p* = 0.026 (Fig. 1A). Additionally, normal and normal pair breast tissues had significantly higher alpha diversity as assessed by Richness (*p* = 0.017), Chao1 (*p* = 0.0021), and Fisher’s alpha (*p* = 0.00087) metrics, compared with breast tumor tissue (Fig. 1A). To determine differences in beta diversity, we visualized the overall differences between the microbiome profiles of the three groups using Principal Coordinate Analysis (PCoA) of unweighted UniFrac distances (Fig. 1B). We observed that normal samples clustered significantly different than tumor samples (Adonis: R^2^ = 0.039, *p* = 0.002, 999 permutations). Normal and adjacent normal tissue samples displayed greater dissimilarity along PC2 (5.54%) compared with breast tumor tissues. Supplementary Table 2 further illustrates differences in alpha and beta diversity and analysis of similarities (ANOSIM) between two comparison groups.

**Figure 2:**
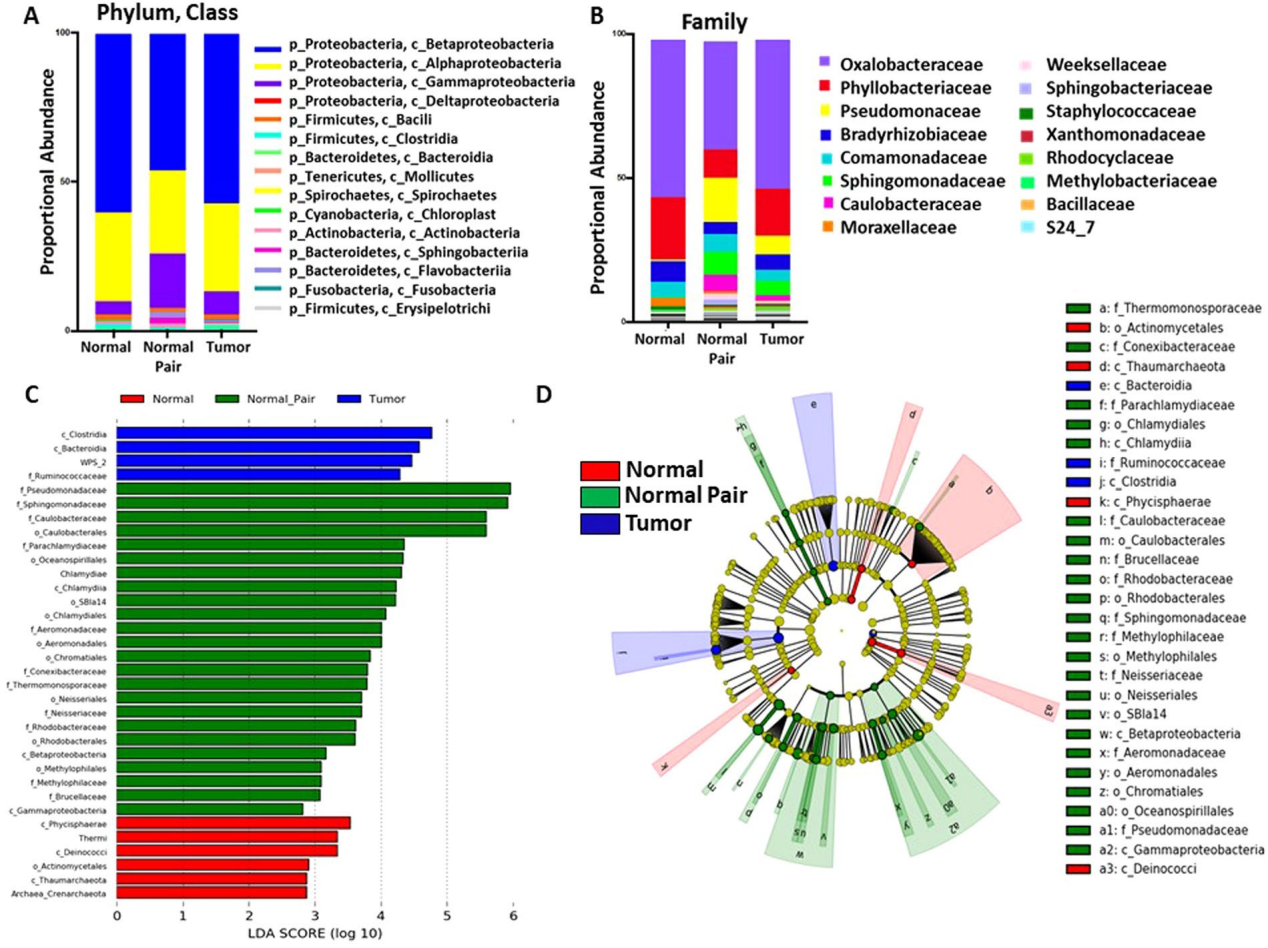
Breast microbiota are distinct between normal, normal pair, and tumor breast tissues. (**A**) Taxonomic profiles of normal (n = 8), normal pair (n = 11), and breast tumor tissue (n = 64) microbiota at phylum level and (**B**) family level for taxa with a relative abundance >0.5% are shown. (**C**) Linear Discriminate Analysis (LDA) scores predict microbiota associated with normal (n = 8), normal pair (n = 11), and breast tumor tissue (n = 64) microbiomes. (**D**) Circular cladogram of differentially abundant taxa increased in normal (n = 8), normal pair (n = 11), and breast tumor (n = 64) tissues. Each letter in concentric ring of nodes represents a taxonomic rank by either class, order, or family. Black arrows indicate taxa identified as significantly increased in normal tissues as compared to breast tumors.

The microbial composition between normal, normal pair, and tumor tissues differed at the phylum, class, and family levels (Fig. 2A,B). At the phylum level, Proteobacteria (classes Betaproteobacteria, Alphaproteobacteria, and Gammaproteobacteria) dominated in the breast, followed by Firmicutes, Bacteroidetes and Actinobacteria, in that order (Fig. 2A). At the family level, *Oxalobacteraceae* in phylum Proteobacteria, dominated in normal, normal pair, and tumor tissues. Additionally, the relative abundance of family *Pseudomonadaceae* (phylum Proteobacteria), a microorganism implicated in antibiotic resistance^14^, was higher in normal pair and tumor breast tissues as compared to normal tissues (Fig. 2B). To further evaluate microbiome differences between normal, normal pair, and tumor breast tissues, Linear discriminant analysis Effect Size (LEfSe) analysis was used to discover different compositions of microbiota and to identify significant cancer-associated biomarkers (Fig. 2C,D). Class Clostridia, Bacteroidia, WPS_2, and family *Ruminococcaceae* was most abundant in tumor samples (Linear Discriminant Analysis (LDA > 4) while families specific to phylum Proteobacteria: *Pseudomonadaceae*, *Sphingomonadaceae*, and *Caulobacteraceae* (LDA > 5) was abundant in normal pairs (LDA > 5) (Fig. 2C,D).

**Figure 3:**
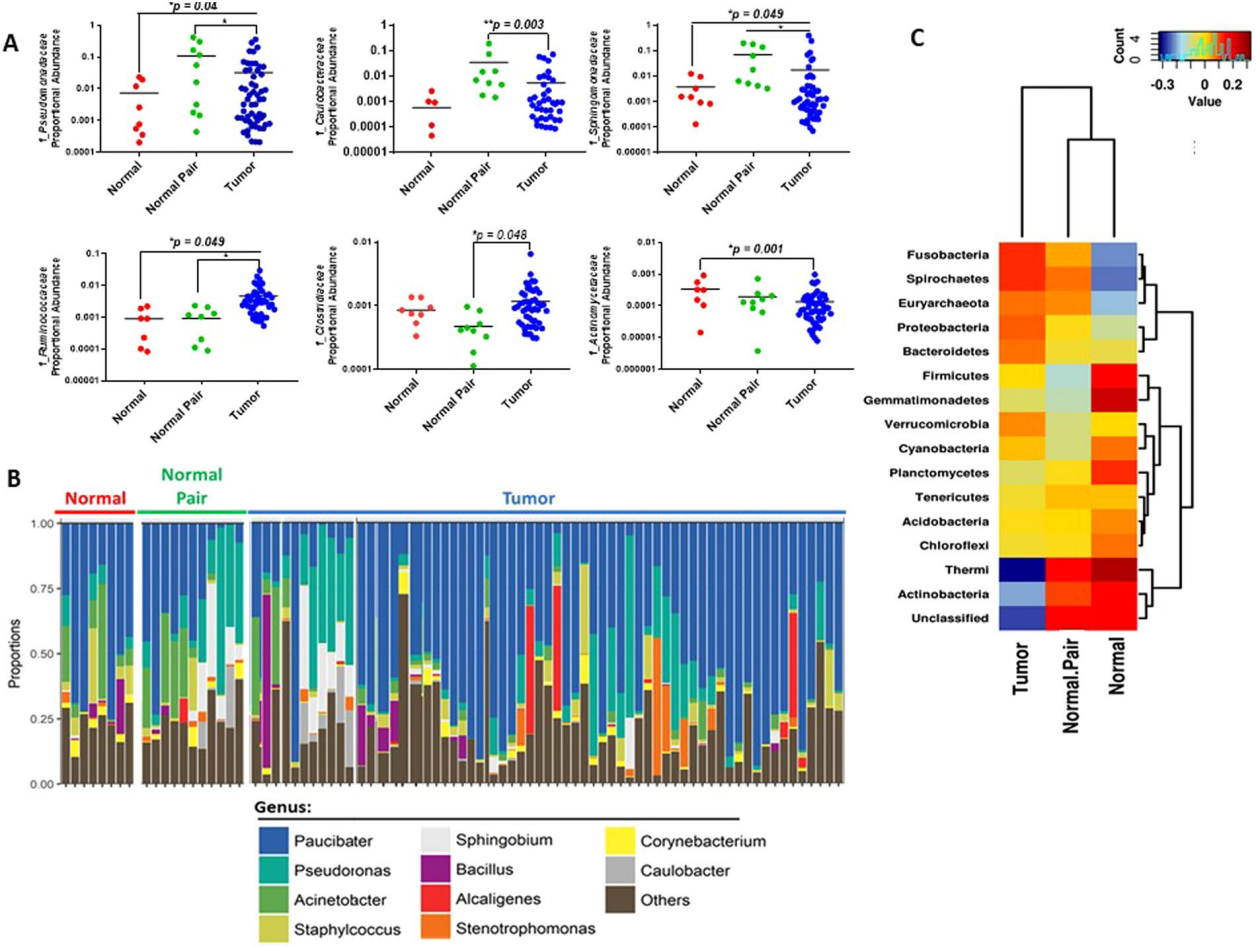
Proportional abundances of microbiota families differ between normal and breast tumor tissues. (**A**) Individual differences in proportional abundances of the most significantly altered microbial families between normal (n = 8), normal pair (n = 11), and breast tumor (n = 64) tissues. Shown are scatter plots of the mean +/− standard error measurement. A *p* < 0.05 is considered statistically significant. (**B**) Bar plots illustrating the relative abundances of genus level microbiota in normal (n = 8), normal pair (n = 11), and breast tumor (n = 64) tissues. (**C**) Spearman heatmap illustrating levels of phyla in breast tumors by tissue type. Blue color represents rare or absent phyla while red color represents abundant phyla. Hierarchical clustering of phyla and sample types are displayed as dendrograms.

We further examined inter-individual differences in abundance of microbiota in normal, normal pair, and tumor breast tissues at the family (Fig. 3A) and genus level (Fig. 3B). At the family level, *Pseudomonadaceae* (phylum *Proteobacteria*), *Sphingomonadaceae* (phylum Bacteroidetes), and *Ruminococcaceae* (phylum Firmicutes) was significantly lower in normal tissues as compared to tumor tissues whereas *Actinomycetaceae* (phylum Actinobacteria) was significantly higher in normal breast tissues (Fig. 3A). By contrast, the relative abundance of family *Ruminococcaceae* and *Clostridia* (phylum Firmicutes) was significantly lower in normal pair tissues as compared to breast tumor tissues. Inter-individual differences in relative abundance of bacteria was also evident at the genus level between normal, including normal pairs, and breast tumor tissues (Fig. 3B). We further generated a Spearman heatmap to visualize all phyla levels across normal, normal pair and tumors (Fig. 3C). This analysis demonstrated fewer Thermi and Actinobacteria and elevated Fusobacteria and Spirochetes in tumor tissues than in non-tumor tissue samples.

**Figure 4:**
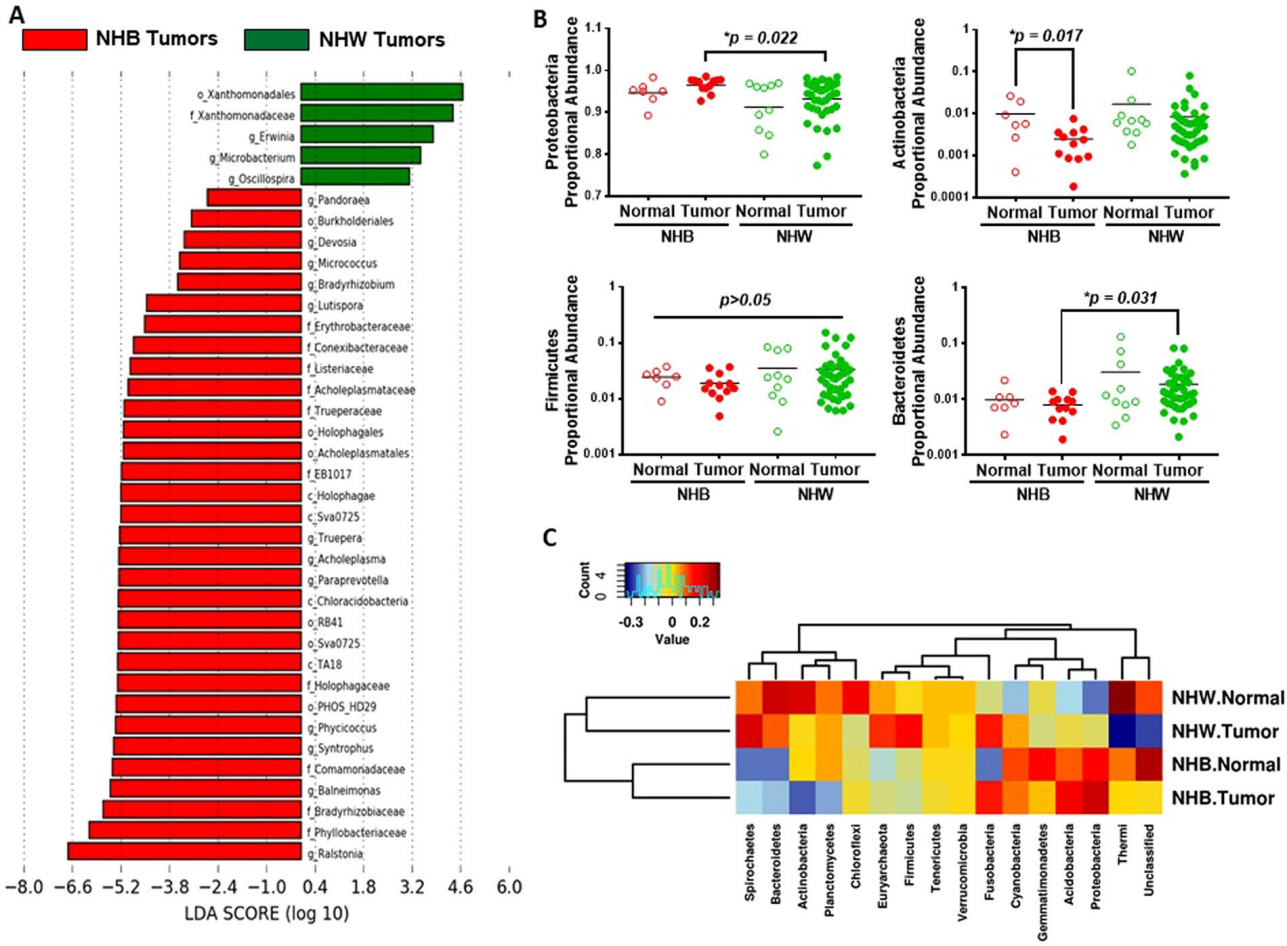
Proportional abundances of breast microbiota differ by race. (**A**) LDA scores were computed for the most differential microbiota abundance between breast tumors by race. (**B**) Scatter plots illustrating the proportional abundance of the four major phyla (*Proteobacteria*, *Actinobacteria*, *Firmicutes*, and *Bacteroidetes*) between normal (n = 19, including normal pairs) and breast tumor (n = 64) tissues of AA (and EA women. A *p* < 0.05 is considered statistically significant. (**C**) Spearman heatmap illustrating levels of phyla in breast tumors between NHB and NHW women. Blue color represents rare or absent phyla while red color represents abundant phyla. Hierarchical clustering of phyla and sample types are displayed as dendrograms.

We next stratified normal and tumor samples to assess microbial differences between NHB and NHW women (Fig. 4). The differences in the quantity of bacteria at the order, family, and genus level between NHB and NHW breast tumors are shown in Fig. 4A. Family *Xanthomonadaceae* (LDA > 4) was most abundant in breast tumors of NHW women, whereas genus *Ralstonia* (both phylum Proteobacteria) was most abundant in breast tumors of NHB women (Fig. 4A). We also observed significant inter-individual differences in relative abundance of phylum Actinobacteria among breast tumors of NHB women as compared to normal breast tissues from NHB women (Fig. 4B). Phylum Bacteroidetes was significantly lower among NHB breast tumors as compared to NHW breast tumors (Fig. 4B). Finally, a Spearman heatmap illustrated the relative abundance of phyla among normal and breast tumors of NHB women compared to NHW women (Fig. 4C).

### TNBC tumors are more abundant in Firmicutes compared to other breast tumor subtypes

**Figure 5:**
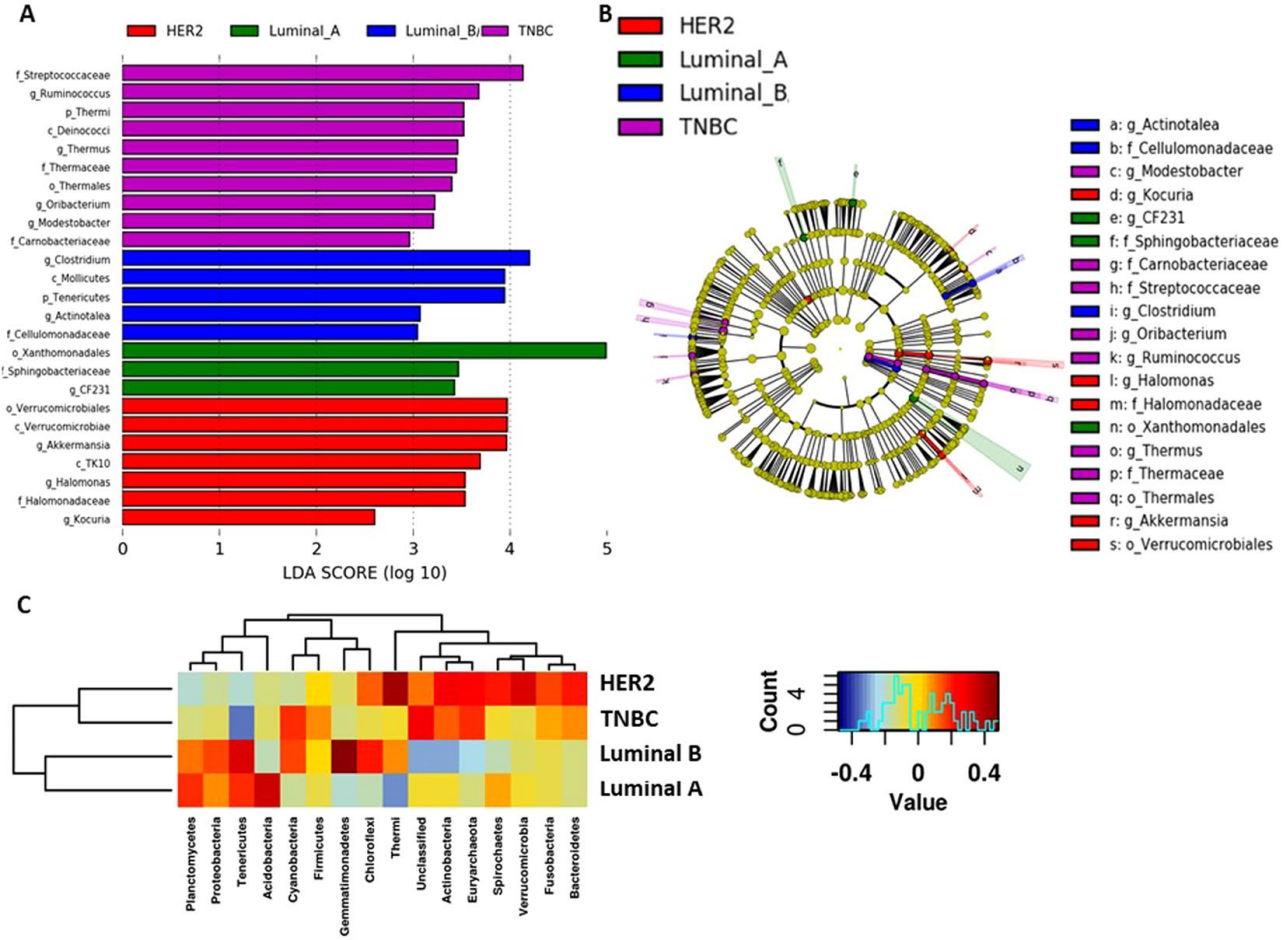
Breast tumor subtypes reveal microbial differences. (**A**) LDA scores were computed for abundance between breast tumor subtypes. (**B**) Circular cladogram reporting taxa consistently differential among the different breast tumor subtypes detected using LEfSe. Colors indicate the group and letters represent the taxa in which each differential clade was most abundant. A *p* < 0.05 is considered statistically significant. (**C**) Spearman heatmap illustrating levels of phyla in breast tumors by subtype. Blue color represents rare or absent phyla while red color represents abundant phyla. Hierarchical clustering of phyla and sample types are displayed as dendrograms.

In order to determine if microbial differences exist between the four major breast tumor subtypes, LEfSe analysis was used to discover different compositions of microbiota and identify significant breast tumor subtype-associated biomarkers (Fig. 5). TNBC was more abundant in genus *Streptococcaceae*, and *Ruminococcus* (both phylum *Firmicutes*) (LDA > 3.5) (Fig. 5A,B). Luminal B tumors were most abundant in genus *Clostridium* (phylum Firmicutes). Luminal A tumors were most abundant in order Xanthomonadales (phylum Proteobacteria) (LDA > 5). HER2 tumors were abundant in genus *Akkermasia* (phylum Verrucomicrobia) (LDA = 4). A Spearman heatmap demonstrated phyla level changes across HER2, luminal A, luminal B, and TNBC tumor subtypes (Fig. 5C), where luminal subtypes demonstrated greater Tenericutes, Proteobacteria, and Planctomycetes phyla. HER2 breast tumors demonstrated greatest abundance of phyla Thermi and Verrucomicrobia while TNBC tumors demonstrated the highest total abundance of phyla Euryarchaeota, Cyanobacteria, and Firmicutes.

**Figure 6:**
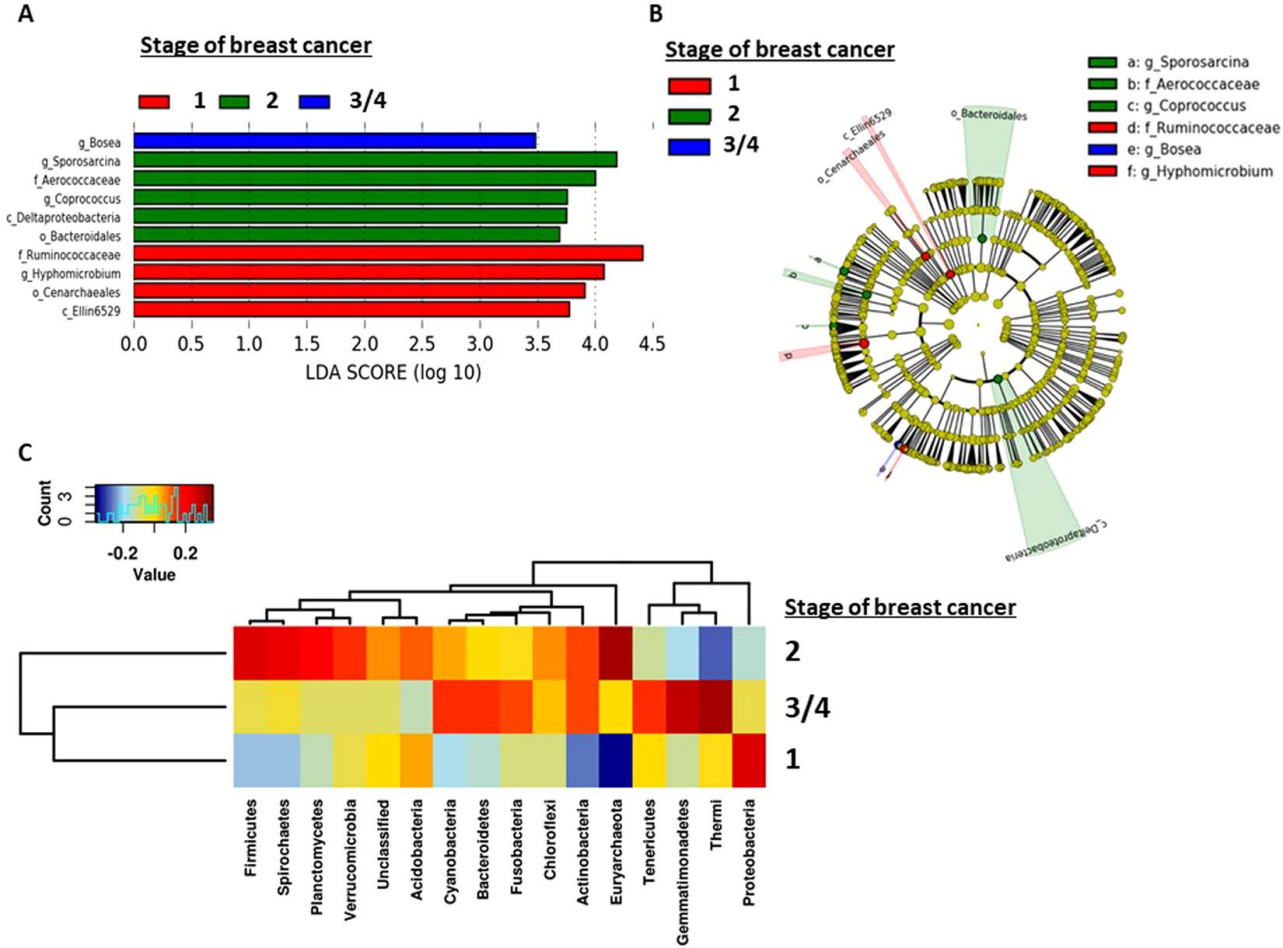
Breast microbiota are different by stage of breast cancer. (**A**) LDA scores were computed for abundance between stages of breast cancer. (**B**) Circular cladogram reporting taxa consistently differential among the different stages of breast cancer detected using LEfSe. Colors indicate the group and letters represent the taxa in which each differential clade was most abundant. A *p* < 0.05 is considered statistically significant. (**C**) Spearman heatmaps illustrating levels of phyla in breast tumors by stage. Blue color represents rare or absent phyla while red color represents abundant phyla. Hierarchical clustering of phyla and sample types are displayed as dendrograms.

Lastly, we determined the extent to which microbiota may be associated with stage of breast cancer (Fig. 6). Family *Ruminococcaceae* (phylum Firmicutes), and genus *Hyphomicrobium* (phylum Proteobacteria) were abundant in stage 1 breast tumors (Fig. 6A,B). Stage 2 breast tumors contained increased genus *Sporosarcina* (phylum *Firmicutes*). Stage 3 and 4 breast tumors showed abundance of only genus *Bosea* (phylum *Proteobacteria*) (Fig. 6A,B). At the phylum level, Spearman heatmap demonstrated elevated Proteobacteria in stage 1; Euryarchaeota, Firmicutes, and Spirochaetes in stage 2, and elevated Thermi, Gemmatimonadetes, and Tenericutes in stage 3/4 (Fig. 6C). Phylogenetic Investigation of Communities by Reconstruction of Unobserved States (PICRUSt) analysis revealed enrichment in photosynthesis proteins in stages 3 and 4 tumors while stage 1 tumors were enriched in energy metabolism, fat digestion and absorption and stage 2 tumors are enriched in phosphotransferase system proteins (Supplementary Fig. S1). These findings suggest that abundance and composition of certain microbiota may be associated with breast tumor subtype and stage of breast cancer.

## Discussion

It is well documented that NHB women are more likely to be diagnosed with TNBC and are most likely to die from breast cancer compared to all other ethnic groups^2,4,12,15,16^. However, NHB women are underrepresented in breast cancer research studies^17,18^, including studies investigating the breast microbiome. Our study is the first to show differences in the breast tissue microbiota between NHB and NHW women. Specifically, we observed that NHB tumors were most enriched in genus *Ralstonia* while NHW tumors were most enriched in order Xanthomonadales, both belong to phylum Proteobacteria. Constantini *et al*. were the first to observe the presence of *Ralstonia* in core needle biopsy breast tissue^19^. There has also been a correlation between the presence of *Ralstonia* and most cancer types, including breast cancer^20^. Thus, it is plausible that enrichment of *Ralstonia* could be a marker of carcinogenesis.

Within the last five years, a wealth of knowledge has been gained from studies examining microbial dysbiosis in breast tissues of women with and without breast cancer^6,9,10,19,21–25^. One of the first published studies on the microbiota of breast cancer tissues was by Xuan *et al*. who identified an association between microbial dysbiosis and breast cancer using next-generation sequencing on DNA isolated from breast tumor tissue and paired normal adjacent tissue from the same patient^21^. Likewise, Hieken *et al*. reported differentially abundant taxa from the phyla Firmicutes, Actinobacteria, Bacteroidetes, and Proteobacteria in breast and skin tissues^9^. Using 16S rRNA sequencing on DNA isolated from breast tissues of women of European descent with and without breast cancer, Urbaniak *et al*. reported that *Bacillus*, *Staphylococcus*, *Enterobacteriaceae* (unclassified), *Comamonadaceae* (unclassified), and Bacteroidetes (unclassified) was most abundant in Canadian and Irish breast cancer patients^10,22^. Similar findings have been observed in tumors and paired normal tissues obtained from Mediterranean women^19^ and Chinese women^6^. The largest breast cancer microbiome study to date was from The Cancer Genome Atlas (TCGA) where RNA sequencing was used to comprehensively analyze the microbiome of 668 breast tumor tissues and 72 non-cancerous adjacent tissues^25^. Unfortunately, these investigations lack inclusion of women of primarily African descent.

Another major finding from our study was the identification of distinct microbiota associated with tumor subtypes and early stage versus advanced stages of breast cancer. Specifically, we observed that TNBC tissues was abundant in family *Streptococcaceae* (LDA > 4). Hermansson *et al*. identified an increased abundance of *Streptococcaceae* in breast milk but the authors caution that *Streptococcaceae* may have originated from the maternal skin or even the environment^26^. Another study found a strong association between OTUs in *Streptococcaceae* and obesity in approximately 600 American adults^27^. Collectively, these findings suggest that *Streptococcaceae* may be associated with increased TNBC risk in obese women. However, to date, only one study described a distinct microbial profile specific for TNBC^23^. In that study, Banerjee *et al*. screened 100 formalin-fixed paraffin embedded archival TNBC samples using a pan-pathogen array chip technology to identify select microbes in TNBC FFPE archival tissues^23^. In contrast to our findings, they detected *Prevotella* at the highest level in TNBC. The discrepancy in enrichment of bacteria in TNBC identified in our study and that of Banerjee *et al*. may be due to the lack of bacterial probes to detect *Streptococcus* bacteria, potential bacterial contamination due to retrospective collection of non-sterile FFPE archival breast tissues, different racial composition, and regional/dietary differences not accounted for.

In addition to breast tumor subtype, we found that stage 1 breast tumors were abundant in all four major phyla whereas stage 2 breast tumors appeared to be less diverse, and stages 3 and 4 breast tumors were abundant in only one bacteria genus: *Bosea*, which belongs to phylum Proteobacteria. Unfortunately, little is known regarding the role of *Bosea* in human disease. However, Heiken *et al*. also observed that malignancy correlated with abundant in taxa of lower abundance including the genera *Fusobacterium*, *Atopobium*, *Gluconacetobacter*, *Hydrogenophaga* and *Lactobacillus* that are members of the phyla Fusobacteria, Actinobacteria, Proteobacteria, and Firmicutes, respectively^9^. By contrast, Meng *et al*. found that the relative abundance of genus *Agrococcus*, which belongs to phylum Actinobacteria, increased with the development of malignancy^6^. Further studies are warranted to determine whether these microbiome signatures can be used as potential biomarkers for predicting tumor subtype, stage of breast cancer and disease progression.

Although a limitation of our study is the small sample size and limited demographic data on obesity status and dietary consumption, which are known to influence the gut microbiota and disease development^28,29^, we were able to identify distinct differences in the microbiota by race, tumor subtype, stage of breast cancer, and disease status. We further validated the presence of the four major breast tissue phyla in our study using 16S rRNA gene sequencing.

In an attempt to reproduce microbiome studies, efforts must be made to preserve the integrity and minimize contamination of surgical breast tissue specimens by immediately snap freezing the tissue in liquid nitrogen and storing long-term at −80 °C to limit exposure to the environment since microbial characteristics can change rapidly with environmental conditions^6,30^. Additionally, protocols need to be standardized and appropriate cross-protocol controls, including DNA extraction reagents, should be included to identify differences in environment-specific contamination, nucleotide extraction, and bioinformatic classification^31^. Collectively, our findings highlight that disease state, tumor subtype, race, and stage of breast cancer display an altered microbiome and should be measured in all breast cancer studies using shotgun metagenomics to reveal deeper characterization and identification of a larger number of microbial species^32^.

In conclusion, progress has been made to support the existence of a breast tissue microbiome. Yet, we have only begun to scratch the surface on to what extent microbial dysbiosis may be linked to breast cancer risk. Further research is needed to understand how microbial dysbiosis influences breast cancer development and treatment outcomes, particularly among ethnically diverse populations who suffer disproportionately from breast cancer health disparities.

## Materials and Methods

### Tissue collection and processing

Fresh, snap frozen aseptically obtained surgical breast tissue specimens from NHB and NHW women (ages 18 to 90 years) with and without breast cancer and clinical information was obtained from the Cooperative Human Tissue Network (Birmingham, AL). Written informed consent was obtained from each participant prior to obtaining breast tissues from the Cooperative Human Tissue Network. Upon receipt, breast tissue samples (n = 83) were immediately stored and maintained at −80 °C until further processing. A total of 19 breast tissues were surgically obtained from NHB women, 62 total breast samples from NHW women, and race/ethnicity was unknown for 1 breast cancer patient who provided both a surgical breast cancer tissue sample and pathologically normal adjacent (normal pair) breast tissue sample. Women free of disease (Normal) underwent reduction mammoplasty for macromastia and their breast tissues were aseptically collected in the operating room. Breast cancer (Tumor) and adjacent normal breast tissue pairs (Normal Pair) from the same donor (n = 11) were also included in this study for comparison. The tissue immediately adjacent (up to 5 cm) to the collected breast cancer tissue sample was evaluated and confirmed by a pathologist to be histologically free of any tumor cells or lesions. Pathological data about the donor breast tissue specimen, including hormone receptor status and grade and stage of breast cancer was obtained from pathological reports. This study was conducted in accordance with the Declaration of Helsinki, and the protocol was approved by the Institutional Review Board of the University of Tennessee Health Science Center (IRB #16-04717-NHSR).

### DNA extraction and 16s rRNA gene sequencing

DNA was isolated from breast tissues under a sterile lamina flow hood using the Qiagen DNA Isolation kit (Qiagen) and quantified by Nanodrop and Quant-iT PicoGreen dsDNA Assay Kit (Invitrogen). Blank controls were used for quality control. DNA was placed into a MoBio PowerMag Soil DNA Isolation Bead Plate. DNA was extracted following MoBio’s instructions on a KingFisher robot. Bacterial 16S sequencing was performed by Microbiome Insights, Vancouver, Canada. Bacterial 16S rRNA genes were PCR-amplified with dual-barcoded primers targeting the V4 region, as per the protocol of Kozich *et al*. (2013). Amplicons were sequenced with an Illumina MiSeq using the 250-bp paired-end kit (v.2). Sequences were denoised, taxonomically classified using Greengenes (v13_8) as the reference database, and clustered into 97%-similarity operational taxonomic units (OTUs) with QIIME (Quantitative Insights into Microbial Ecology) 1.9.1. The potential for contamination was addressed by co-sequencing DNA amplified from specimens and from four each of template-free controls and DNA extraction kit reagents processed the same way as the specimens. Two positive controls, consisting of cloned SUP05 DNA, were also included (number of copies = 2*10^6). Operational taxonomic units were considered putative contaminants (and were removed) if their mean abundance in controls reached or exceeded 25% of their mean abundance in specimens.

### 16S statistical analysis

Following filtering, samples were rarified to a depth of 4000 sequences. Principal Coordinate (PC) analyses were based on unweighted UniFrac distances using even OTU samples, and were generated in EMPeror^33^. Variation in community structure was assessed with permutational multivariate analysis of variance using distance matrices (ADONIS) with treatment group as the main fixed factor and using 999 permutations for significance testing. We used linear discriminant analysis of effect size (LEfSe) to test for significance and perform high-dimensional biomarker identification^34^. Raw OTU tables were imported into Calypso 8.84 for further analysis, including alpha diversity and Spearman heatmaps^35^. Alpha diversity and richness were estimated with the Shannon index, Chao1 (estimator of abundance), Fisher’s alpha, and Richness metrics. Spearman’s heatmaps were calculated based on phyla level abundances with sample and taxa clustering displayed with dendrograms. The significance of diversity differences was tested with one-way ANOVA. The calculation of *P*-values was done with Mann Whitney *t*-test.

## Supplementary information

supplementary file

Dataset 1

## Data Availability

Raw microbiome data analyzed for the current study is provided in supplementary files.
