# Supplementary material for: Distinct microbial communities that differ by race, stage, or breast-tumor subtype in breast tissues of non-Hispanic Black and non-Hispanic White women
Source: Sci Rep. 2019 Aug 16;9:11940. doi: 10.1038/s41598-019-48348-1 (PMC6697683; doi:10.1038/s41598-019-48348-1)
Supplement: Supplementary file 1 — supplementary file [file 41598_2019_48348_MOESM1_ESM.pdf]

Distinct microbial communities that differ by race, stage, or breast-tumor subtype in breast tissues of non-Hispanic Black and non-Hispanic White women

Alana Smith[1±], Joseph Pierre[2±], Liza Makowski[3,4], Elizabeth Tolley[5], Beverly Lyn-Cook[6], Lu Lu[1], Gregory Vidal[7], and Athena Starlard-Davenport[1*]

[1]Department of Genetics, Genomics and Informatics, [2]Pediatrics, [3]Medicine, [4]Pharmaceutical Sciences, [5]Preventive Medicine, University of Tennessee Health Science Center, Memphis, TN 38133, USA, [6]Division of Biochemical Toxicology, FDA/National Center for Toxicological Research, Jefferson, AR, [7]West Cancer Center, Memphis TN 38138

No conflicts

[±] Equal first authors

***Correspondence to:**

Athena Starlard-Davenport, PhD
Department of Genetics, Genomics and Informatics
University of Tennessee Health Sciences Center
71 S Manassas, Room 419
Memphis, TN 38163
Phone: 901-448-3085
Fax: 901-448-3500
E-mail: astarlar@uthsc.edu

Supplementary Table S1. Clinicopathological features of breast tissue specimens analyzed in this study

Sample	age	race	Menopausal status	Stage	Tumor Subtype
**Normal**					
N1	35	W	Pre		
N2	25	B	Pre		
N3	21	W	Pre		
N4	28	W	Pre		
N5	23	B	Pre		
N6	18	B	Pre		
N7	35	B	Pre		
N8	52	B	Post		
**Tumor**					
T1	55	B	Post	III	NA
T2	43	W	Pre	III	Luminal B
T3	49	W	Pre	I	Luminal A
T4	38	W	Pre	III	NA
T5	NA	W	NA	III	NA
T6	60	W	Post	III	TNBC
T7	48	W	Pre	III	TNBC
T8	25	W	Pre	III	Luminal B
T9	NA	W	NA	III	NA
T10	49	W	Pre	I	Luminal A
T11	49	W	Pre	III	Luminal B
T12	31	B	Pre	I	Luminal A
T13	37	B	Pre	I	Luminal A
T14	40	W	Pre	III	TNBC
T15	25	B	Pre	III	Luminal B
T16	53	W	Post	III	NA
T17	44	W	Pre	II	Luminal B
T18	52	B	Post	II	Luminal A
T19	54	B	Post	II	Luminal A
T20	32	W	Pre	III	HER2
T21	55	W	Post	II	Luminal B
T22	50	W	Post	I	Luminal A
T23	59	W	Post	III	TNBC
T24	30	B	Pre	III	HER2
T25	62	W	Post	III	TNBC
T26	55	W	Post	I	Luminal A
T27	46	W	Pre	II	Luminal A
T28	45	W	Pre	II	Luminal A
T29	49	W	Pre	II	Luminal A
T30	52	W	Post	III	Luminal B

T31	43	W	Pre	I	Luminal B
T32	32	W	Pre	III	TNBC
T33	50	W	Post	II	Luminal B
T34	41	W	Pre	II	Luminal A
T35	43	W	Pre	II	Luminal A
T36	46	W	Pre	II	Luminal A
T37	48	W	Pre	II	Luminal A
T38	50	W	Post	III	Luminal B
T39	43	W	Pre	III	Luminal B
T40	48	W	Pre	II	Luminal A
T41	47	W	Pre	III	HER2
T42	53	W	Post	II	Luminal A
T43	53	W	Post	III	TNBC
T44	31	B	Pre	II	Luminal A
T45	31	W	Pre	III	HER2
T46	48	B	Pre	II	Luminal A
T47	52	W	Post	III	Luminal B
T48	40	W	Pre	III	TNBC
T49	66	W	Post	III	TNBC
T50	51	W	Post	III	TNBC
T51	45	W	Pre	II	Luminal B
T52	39	B	Pre	III	Luminal B
T53	52	W	Post	II	TNBC
**Adjacent Normal/Tumor Pairs from Same Patient**					
N/T P1	55	W	Post	III	TNBC
N/T P2	45	U	Pre	III	NA
N/T P3	45	W	Pre	III	HER2
N/T P4	45	W	Pre	II	TNBC
N/T P5	72	W	Post	III	TNBC
N/T P6	59	B	Post	I	Luminal A
N/T P7	41	B	Pre	II	Luminal A
N/T P8	43	W	Pre	II	Luminal A
N/T P9	47	W	Pre	I	HER2
N/T P10	41	W	Pre	III	TNBC
N/T P11	71	W	Post	III	NA

Supplemental Table S2. P-values illustrating differences in alpha and beta diversity between groups

	N vs NP	N vs T	NP vs T
Shannon	0.230	0.470	***0.0071*
Richness	0.370	0.063	**0.012*
Chao1	0.210	0.052	***0.0015*
Fisher's alpha	0.270	**0.036*	****0.0006*
ANOSIM	0.137	0.226	0.366

Abbreviations: N: normal; NP: normal pair; T: tumor
Statistical significance: *p<0.05, **p<0.01, ***p<0.001

Supplementary Figure S1

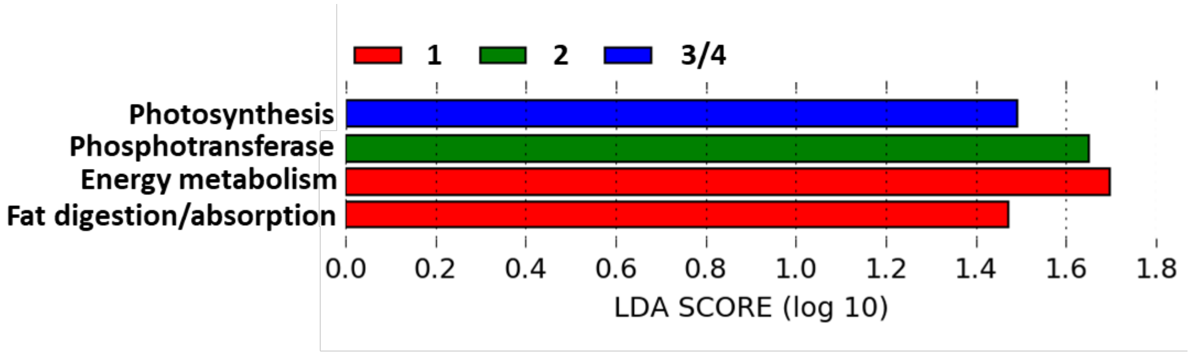

Figure S1. LDA scores predict gene function associated with breast cancer stage using Phylogenetic Investigation of Communities by Reconstruction of Unobserved States (PICRUSt). (Linear discriminant analysis; LDA).
